# Analysis of Newly Identified and Rare Synonymous Genetic Variants in the *RET* Gene in Patients with Medullary Thyroid Carcinoma in Polish Population

**DOI:** 10.1007/s12022-017-9487-2

**Published:** 2017-06-24

**Authors:** Maria Sromek, Małgorzata Czetwertyńska, Magdalena Tarasińska, Aneta Janiec-Jankowska, Renata Zub, Maria Ćwikła, Dorota Nowakowska, Magdalena Chechlińska

**Affiliations:** 1 Department of Immunology, Maria Sklodowska-Curie Institute - Oncology Center, Warsaw, Poland; 2 Laboratory of Cellular Immunology, Maria Sklodowska-Curie Institute - Oncology Center, W.K. Roentgen 5, 02-781 Warsaw, Poland; 3 Department of Nuclear Medicine and Endocrine Oncology, Maria Sklodowska-Curie Institute - Oncology Center, Warsaw, Poland; 4 Department of Oncology, The Children’s Memorial Health Institute, Warsaw, Poland; 5 Department of Diagnostic Laboratory of Genetic Predispositions, Maria Sklodowska-Curie Institute - Oncology Center, Warsaw, Poland; 6 Department of Molecular and Translational Oncology, Maria Sklodowska-Curie Institute - Oncology Center, Warsaw, Poland; 7 Department of Gastroenterological Oncology, Maria Sklodowska-Curie Institute - Oncology Center, Warsaw, Poland; 8 Genetic Counseling Unit, Cancer Prevention Center, Maria Sklodowska-Curie Institute - Oncology Center, Warsaw, Poland

**Keywords:** *RET* gene, Synonymous mutation, Medullary thyroid carcinoma, MEN2, Thyroid

## Abstract

Gain-of-function germline mutations of the *RET* proto-oncogene are responsible for initiation of carcinogenesis within the thyroid gland and development of hereditary form of medullary thyroid carcinoma and MEN2 syndrome. Genotype-phenotype correlations are established for most *RET* mutations, but the importance of the synonymous changes in this gene remains debatable. We aimed to analyze *RET* gene variants in Polish population. Genetic testing for the *RET* gene variants was performed with standard methods in 585 people aged 1–85, including 448 patients with medullary thyroid carcinoma and 131 of their first- and second-degree relatives, as well as six patients suspected of MTC/MEN2. Besides the most frequent synonymous changes, p.Leu769Leu, p.Ser836Ser, and p.Ser904Ser, four rare changes—c.1827C>T (p.Cys609Cys), c.2364C>T (p.Ile788Ile), c.2418C>T (p.Tyr806Tyr), and c.2673G>A (p.Ser891Ser)—were found in the *RET* gene, in the Polish population. Two of the rare changes, p.Cys609Cys and p.Ile788Ile, had not been previously described. The frequency of molecular synonymous variants in the general population was evaluated by testing 400 anonymous blood samples of neonates. Our findings may contribute to a better understanding of the genetic diversity of the *RET* gene and the involvement of synonymous variants in this diversity.

**Electronic supplementary material:**

The online version of this article (doi:10.1007/s12022-017-9487-2) contains supplementary material, which is available to authorized users.

## Background

Proto-oncogene *RET* (rearranged during transfection) encodes a single-pass transmembrane receptor of the tyrosine kinase family. The *RET* gene lies on the chromosome 10q11.2 [1] and comprises 21 exons. RET protein is composed of three domains: an extracellular ligand-binding domain with four cadherin-like repeats and a cysteine-rich region, a transmembrane domain and a cytoplasmic region with the tyrosine kinase domains [2, 3]. Missense, germline gain-of-function mutations in the *RET* proto-oncogene are associated with type 2 multiple endocrine neoplasia syndromes (MEN2A or MEN2B) and familial medullary thyroid carcinoma (FMTC) [4–7]. Medullary thyroid carcinoma (MTC) is a common component of these syndromes. MTC occurs as a part of an inherited disorder (approx. 20–25% of cases) and as a sporadic tumor (the remaining 75% of cases) [8]. The disease phenotypes and the age of onset are associated with codon-specific *RET* mutations and their transforming potential. Considering MTC aggressiveness and the co-existing endocrinopathies such as pheochromocytoma (PHEO), hyperparathyroidoism (HPTH), cutaneous lichen amyloidosis (CLA), and Hirschsprung’s disease (HD), the American Thyroid Association (ATA) Guidelines currently divide germline *RET* mutations into three risk categories: ATA–HST (the highest risk—patients with MEN2B and the *RET* codon M918T mutation), ATA-H (the high risk—patients with *RET* codon C634 mutations and the *RET* codon A883F mutation), and ATA-MOD (moderate risk—patients with all other mutations in the *RET* gene) [9].

Beside the changes of the confirmed pathogenic significance, there are polymorphic changes (frequency: ≥1%) [10] in the *RET* gene. In the European population, the most common polymorphic variants (MAF (*minor allele frequency*) >5%) [11] are the following: c.2307 T>G (p.Leu769Leu) in exon 13 (allele G frequency: 24%); c.2071 G>A (p.Gly691Ser) in exon 11 (allele A frequency: 19%); c.2712 C>G (p.Ser904Ser) in exon 15 (allele G frequency: 19%), and c.2508 C>T (p.Ser836Ser) in exon 14 (allele T frequency: 6%) [12, 13]. Their role in tumorigenesis is still unclear, and there are conflicting data as to whether these changes can modify the risk of developing MTC [14–16]. In addition to these common polymorphic changes in the *RET* gene, there are also rare synonymous or nonsynonymous allelic variants (MAF < 0.5%) of uncertain significance. Identification of these rare changes in the context of specific symptoms of the disease is extremely significant for a better understanding of the role they potentially play in the RET receptor function.

The role of synonymous genetic variants is a matter of a particular controversy. Such changes, according to the Anfinsen’s principle postulating that the amino acid sequence of the protein alone determines the structure and functions of a protein, were for a long time referred to as “silent” [17]. Recent studies have revealed that the synonymous changes may affect the protein function and cause many diseases.

Several mechanisms have been proposed to explain the pathogenic role of synonymous changes in cancer. Synonymous changes can influence post-transcriptional RNA processing [18–24] and post-transcriptional miRNA-dependent regulation, by altering miRNA binding sites [25–31]. At present, a few miRNAs regulating protein RET expression are known [32, 33], all of which bind the 3’UTR region of *RET*. Synonymous changes may affect the global mRNA stability [34–37], or the local stability in the start codon region [38–40], or the maintenance of cell homeostasis [41]. Synonymous changes can also influence the speed and accuracy of translation. One way of kinetic control of translation is codon usage. The synonymous mutation can slow down or accelerate the rate of protein synthesis and lead to protein misfolding. SNPs can generate translation pause sites (ribosome stalling) resulting in alternative conformers during co-translational folding [38, 42].

Some synonymous changes are directly related to the pathogenesis of a disease, e.g., in Treacher-Collins’ syndrome, the synonymous variant c.3612A>C in the *TCOF1* gene causes exon 22 skipping and mis-splicing and results in defective mRNA [43], and in cystic fibrosis, a structural instability of mRNA, caused by the synonymous polymorphism p.Ile507Ile in the context of ΔF508 *CFTR*, could be responsible for the reduced translational rate and lower cellular expression level of CFTR protein [44]. Several synonymous mutations have been shown to be associated with carcinogenesis and influence cancer risk by various mechanisms. For example, the specific silent mutations (p.Pro36Pro) in *TP53* gene lower the affinity of the *TP53* mRNA for the regulatory protein MDM2, and thereby reduce the ability of TP53 to activate apoptosis [45]. The synonymous variant p.Pro72Pro has been associated with an elevated risk of lung cancer [46], and the synonymous changes, rs1061302 and rs709816 in the *NBS1* gene, are linked with smoking-related cancers (lung, larynx, liver, and bladder) [47].

The aim of this study was to examine a few rare synonymous allelic variants of the *RET* gene in MTC patients in Polish population. Some of the variants have not been previously studied in MTC patients.

## Patients

Genetic testing for *RET* mutations was performed in 585 people, aged 1–85 years, including 448 patients with MTC and 131 of their first- and second-degree relatives, and six patients suspected of MTC/MEN2 with other diseases (PTC, PHEO, renal carcinoma, adrenal gland tumor, nodular thyroid disease, and carcinoid of the stomach) (Table 1).

**Table 1:** Patients

Phenotype	No pathogenic changes (*n*)	Pathogenic changes (*n*)
MTC/MEN2A/MEN2B	358	76
MTC + other cancer	8	–
MTC + other thyroid cancer	4	–
MTC/HSCR	–	2
Other	5	1
Asymptomatic	101	30

Most patients and their kindred were taken care of at the Outpatient Clinic of Thyroid Diseases and Genetic Counseling Unit Cancer Prevention Center and the Maria Skłodowska-Curie Memorial Cancer Center and Institute of Oncology in Warsaw between 1998 and 2016. Twenty-nine adolescent patients aged 1–18 had been admitted to other hospitals: the Department of Pediatric Surgery at the Collegium Medicum of Nicolaus Copernicus University in Bydgoszcz, the Department of Oncology at The Children’s Memorial Health Institute in Warsaw, and the Department of Pediatrics and Endocrinology of the Warsaw Medical University. The peripheral blood of adolescent patients was collected for genetic testing in the Maria Sklodowska-Curie Memorial Cancer Center and Institute of Oncology in Warsaw. All patients were subjected to the standard diagnostic procedures, as published by Paszko et al. [48]. Patients with cytologically or histopathologically confirmed MTC were enrolled for the detailed genetic testing. Six exons (10, 11, 13, 14, 15, and 16) of the *RET* gene were analyzed in all patients. In 26 patients, including those at risk of inherited MTC (genetic load in the family), those with aggressive MTC disease, or with the specific MEN syndrome symptoms, as well as those with the early age of onset, additional exons (5, 8, 9, 12, 18, and 19) were sequenced, to check for other mutations in the *RET* gene. The analyzed exons were selected based on the ATA Guidelines Task Force on Medullary Thyroid Carcinoma [49] and ARUP Scientific Resource for Research and Education Mutation Databases [50].

The frequency of molecular synonymous variants in exons 10, 11, 13, 14, and 15 in the general population was evaluated by testing 400 anonymous blood samples of neonates.

## Methods

DNA was extracted from the peripheral blood lymphocytes using a commercial kit *Genomic Midi AX* (Biotechnology). Germline *RET* gene mutations were screened in exons 10, 11, 13, 14, 15, and 16 and additionally in exons 5, 8, 9, 12, 18, and 19 (see Supplementary Material for primer sequences). All the tested fragments of the *RET* gene were amplified using PCR technique. Following purification on *Centri-Sep Spin Columns*, (Applied Biosystems), PCR products were subjected to electrophoresis in a Perkin Elmer ABI Prism Sequencer using fluorescently labeled terminators *BIG DYE v.3.1* (Applied Biosystems). Germline mutations were identified by comparing the sequences of the tested samples with the relevant correct *RET* sequences*:* NM 020630.4. Genotype-phenotype correlations and the identified changes-related risk of aggressive MTC were verified by analyzing public databases [32, 49–51].

## Results

Direct sequencing analysis of the *RET* proto-oncogene in 585 people revealed germline pathogenic mutations in eight exons (10, 11, 13, 14, 15, 16, 18, and 19) in 79 patients (aged 1–75 years) (17.4% of patient group) and their 30 unaffected kindred (aged 1–80 years) (22.9% of asymptomatic group). Three hundred seventy patients (aged 7–85 years) (81.5% of patient group) were diagnosed with sporadic MTC (sMTC) and five patients suspected of MTC/MEN2 with other diseases (1.1% of patient group) (Table 1). No carrier of the pathogenic mutation was found in this group. As revealed by genetic studies of patients and their relatives, individuals of all series presented several synonymous genetic changes in the *RET* gene. Apart from the most frequent polymorphic variants (p.Leu769Leu, p.Ser836Ser, and p.Ser904Ser) [15], four rare synonymous changes were found, and two new changes were identified, c.1827 C>T (p.Cys609Cys) and c.2364 C>T (p.Ile788Ile) (Table 2).

**Table 2:** Synonymous variants in four codons of the *RET* gene and nonsynonymous changes in these codons

Exon	Codon	Genotype (cDNA)	Codon change	Protein change	Families (*n*)	Patients (*n*)	Asymptomatic carriers (*n*)	Phenotype	Average age of MTC	Patients gender F/M	Classification
10	609^a^	*c. 1827 C>T*	*TGC-TGT*	*Cys609Cys*	*1*	*1*	*2*	*MTC*	*12*	*0/1*	*Uncertain significance*
		c. 1825 T>C	TGC-CGC	Cys609Arg	1	0	1	–	–	–	Pathogenic
		c. 1826 G>T	TGC-TTC	Cys609Phe	1	1	0	FMTC/MEN2A	63	1/0	Pathogenic
		c. 1826 G>A	TGC-TAC	Cys609Tyr	1	1	2	FMTC/MEN2A	33	1/0	Pathogenic
13	788^a^	*c.2364 C>T*	*ATC/ATT*	*Ile788Ile*	*1*	*1*	*0*	*MTC*	*46*	*1/0*	*Uncertain significance*
14	806	*c. 2418 C>T*	*TAC/TAT*	*Tyr806Tyr*	*1*	*1*	*0*	*Carcinoid of the stomach/MEN2A susp.*	*50*	*1/0*	*Uncertain significance*
15	891	*c.2673 G>A*	*TCG/TCA*	*Ser891Ser*	*1*	*1*	*0*	*MTC*	*53*	*0/1*	*Uncertain significance*
		*c.2673 G>A* +c.1853G>C,	*TCG/TCA* +TGC/TCC	*Ser891Ser* +Cys618Ser	*1*	*1*	*0*	*FMTC/MEN2A*	*37*	*1/0*	
		c. 2671 T>G	TCG/GCG	Ser891Ala	2	3	0	FMTC/MEN2A	57.3	2/1	Pathogenic

Italic text indicates all rare synonymous changes found in the study population

^a^Codons of the *RET* gene with newly identified synonymous changes

## Patients Report

### Genetic Variant in Exon 10: c.1827 C>T (p.Cys609Cys)

A 10-year-old male patient with no family history of MEN syndrome, familial MTC, or sporadic MTC was diagnosed with tumor (16 × 22 × 30 mm) located in the right lobe of the thyroid. The level of serum calcitonin was markedly elevated to 991 pg/mL. The results of abdominal cavity ultrasound and chest x-ray imaging were negative. The patient underwent total thyroidectomy, cervical lymph node dissection, and partial removal of the thymus. The tumor was classified as a monofocal medullary thyroid carcinoma (pT2N1aM0). The postoperative calcitonin level was 5.57 pg/mL. There were no other symptoms of MEN2 syndrome. Two years after the surgery, control studies showed an increase in the level of calcitonin (36.7 pg/mL) and an enlargement of the cervical lymph node. The lymph node was removed, and further examinations (USG and PET-CT results, serum calcitonin level of 24 pg/mL, and CEA level of 2 ng/mL) showed no recurrence.

**Fig. 1:**
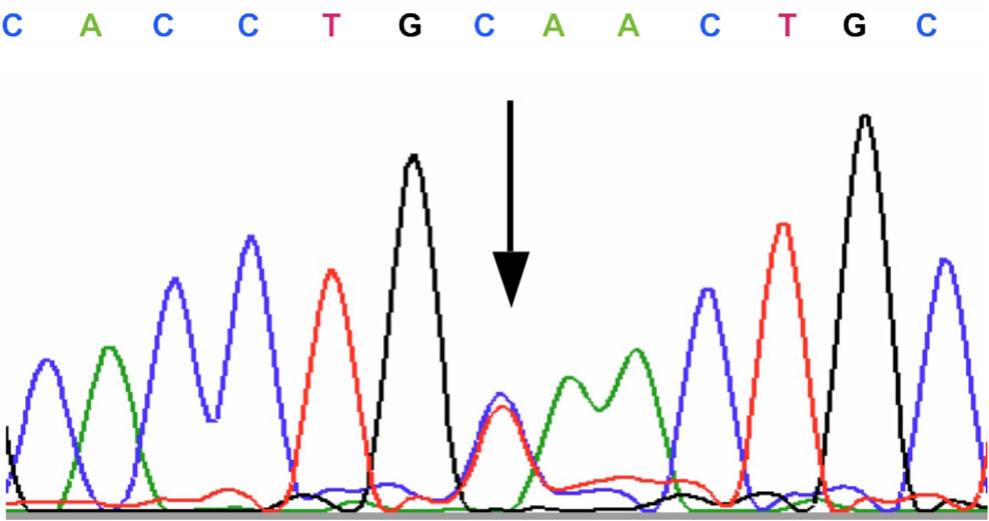
A synonymous variant c.1827 C>T (p.Cys609Cys) in exon 10 of the *RET* gene

Genetic testing revealed a synonymous variant: c.1827 C>T (p.Cys609Cys) in exon 10 (Fig. 1) (Table 2). No germline mutations in the remaining exons (5, 8, 11, 12, 13, 14, 15, 16, 18, and 19) were found. In exon 13 of the *RET* gene, only one heterozygous polymorphic change, p.Leu769Leu, was found.

**Fig. 2:**
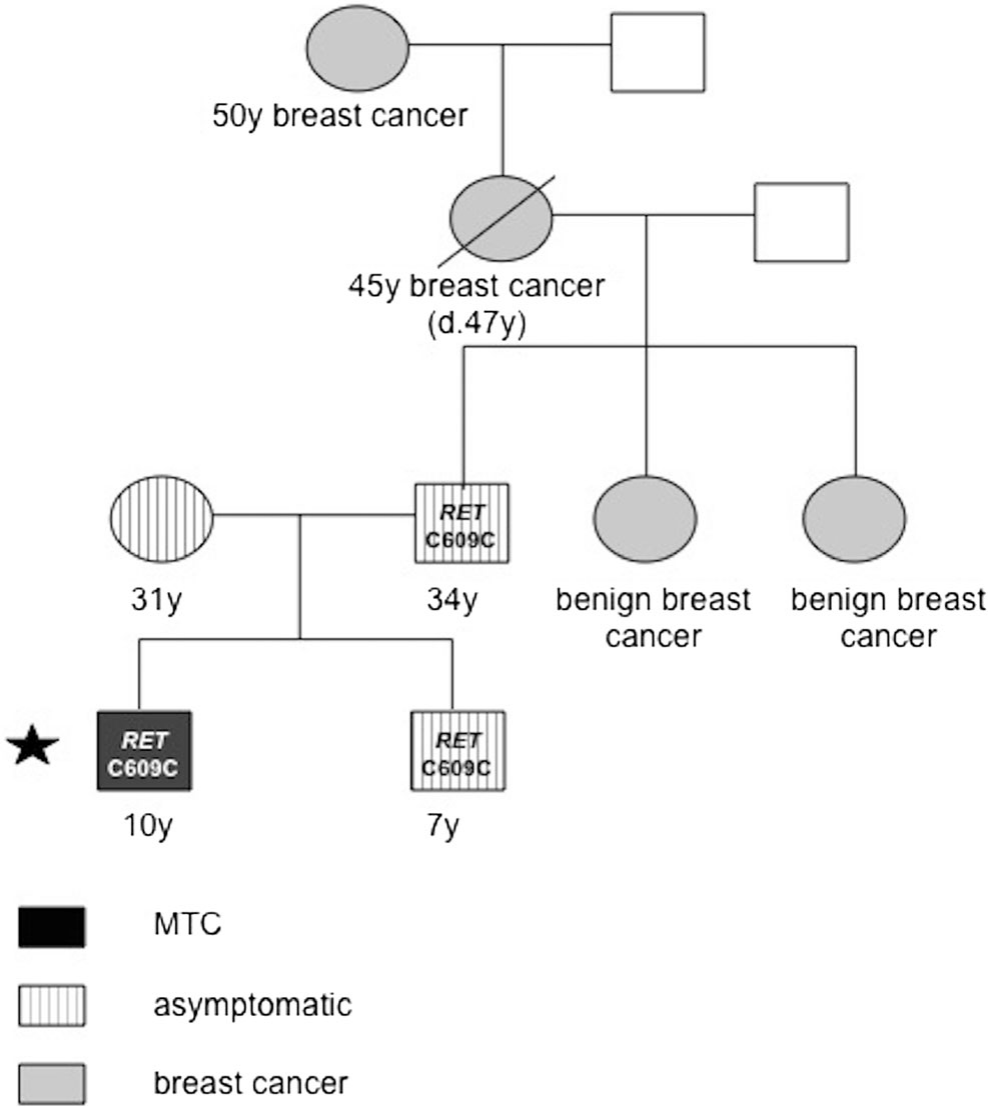
Pedigree of the patient’s family. The probant is indicated by the *black asterisk*

Genetic analysis of the *RET* gene in patient’s relatives (parents and younger brother) revealed the same synonymous change in exon 10, in the father and the brother (Fig. 2). Due to the family history of cancer, four additional genes, BRCA1, BRCA2, CHEK2, and NBS1, were tested in the father of the patient, and no mutations were found. Neither the father nor the younger brother experienced any symptoms of cancer, as assessed by laboratory tests for serum CEA, calcitonin level, neck, and abdominal ultrasound scanning.

### Genetic Variant in Exon 13: c.2364 C>T (p.Ile788Ile)

**Fig. 3:**
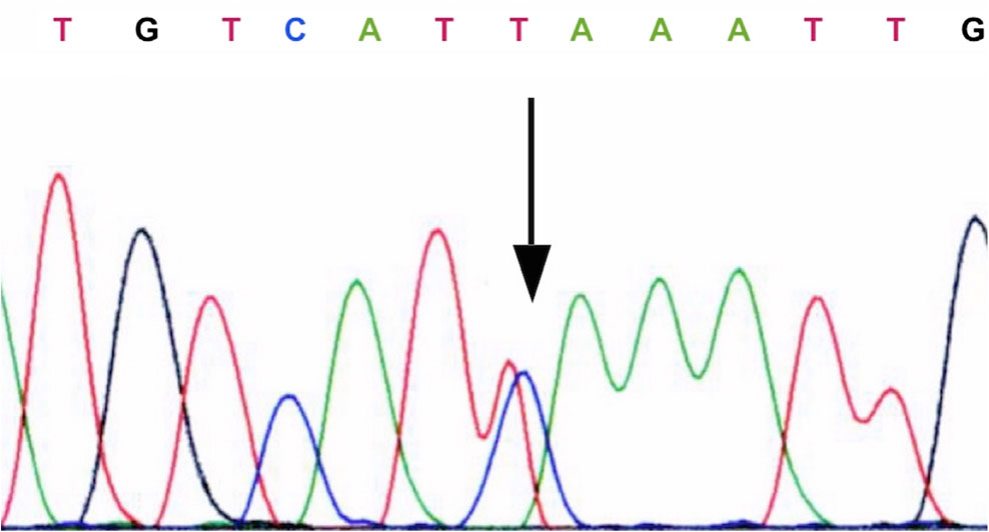
A synonymous variant c. 2364 C>T (p.Ile788Ile) in exon 13 of the *RET* gene

A female carrier of synonymous heterozygous change in exon 13: c.2364 C>T (p.Ile788Ile) (Fig. 3) was diagnosed with sporadic MTC at the age of 46; a tumor of 27 × 36 × 39 mm was located in the left lobe of the thyroid. Serum calcitonin level was elevated to 885 pg/mL. The patient, diagnosed with metastases to the regional cervical lymph nodes, underwent total thyroidectomy, with removal of the central and left lateral lymph node. Apart from the three heterozygous polymorphisms—p.Gly691Ser, p.Leu769Leu, and p.Ser904Ser—no other mutations in the 12 studied exons of the *RET* gene were found (Table 2).

### Genetic Variant in Exon 14: c.2418 C>T (p.Tyr806Tyr)

Because of the nodular thyroid and a 13-mm tumor of the left adrenal gland, and a suspicion of MEN2 syndrome, genetic analysis of the *RET* gene was also performed in 50-year-old female patient with carcinoid of the stomach. There were no pathogenic mutations in the 12 examined exons of the *RET* gene. Only two heterozygous polymorphic changes in exons 13 and 15, p.Leu769Leu and p.Ser904Ser, and one rare synonymous heterozygous change in exon 14, c.2418 C>T (p.Tyr806Tyr), rs553418132, were found (Table 2).

### Genetic Variant in Exon 15: c.2673G>A (p.Ser891Ser)

In two MTC patients, a rare synonymous change, c.2673G>A (p.Ser891Ser), rs201612214, was identified (Table 2). One patient, a 37-year-old woman with bilateral tumors of the thyroid gland (10 × 6 × 23 mm in the right lobe and 24.5 × 19.5 × 33.5 in the left lobe), carried an additional pathogenic mutation, p.Cys618Ser in exon 10, and two polymorphic changes, p.Gly691Ser and p.Ser904Ser. It was not possible to find out whether these genetic variants were cis- or trans-changes. The other, a 53-year-old man with a tumor located in the left lobe of the thyroid gland (7 mm in diameter, pT1a), carried polymorphic variants p.Gly691Ser/p.Ser904Ser of the *RET* gene.

None of the four rare synonymous changes in exons 10, 13, 14, and 15 were found in the general population group.

## Discussion

To assess the significance of rare genetic changes, apart from clinical data, it is necessary to collect information on the carriers of the identified gene changes, their penetration in the family, and their frequency in the general population. We present here several rare genetic variants of the *RET* gene. We also identified two synonymous variants, p.Cys609Cys and p.Ile788Ile, that have not been identified and described before.

In 10–15% of MEN2A and FMTC cases, codons 609, 611, 618, and 620 are affected [52]. Our previous studies have shown that in patients with MTC in Polish population, pathogenic changes occur most frequently in exon 10 of the *RET* gene (38.8% of all mutation), while for example, the frequency of mutations in codon 634 was only 26.8% [48]. More than 60% of mutations in cysteine codons of exon 10 occur in FMTC and 10–15% in MEN2A [7, 53, 54]. All these mutations are associated with a moderate risk of aggressive MTC (ATA-MOD). The most frequent mutations in codon 609 are changes of cysteine into R, G, Y, S, F, and W [50]. However, as different amino acid substitutions of cysteine result in a comparable transforming activity, and it is suggested that the activity depends on the position of the cysteine mutations rather than on the substituting amino acid [14, 55]. So far, no synonymous change in codon 609 has been described. Generally, synonymous changes in exon 10 are rare. The germline synonymous genetic variants have been reported in nine codons (588, 591, 594, 601, 608, 619, 620, 621, and 622) of exon 10, so far. The frequencies of these changes are very low [12, 13].

The question whether the discovered substitution of cysteine to cysteine in codon 609 may be involved in the pathogenesis of MTC remains open. The 34-year-old father of the patient with MTC was an unaffected carrier of the same variant (Fig. 3). However, due to a very young age of onset and the lack of other known pathogenic mutations in all the examined exons of the *RET* gene, the role of this change in the pathogenesis of MTC cannot be ignored.

The other new genetic variant that we found was a synonymous substitution of isoleucine to isoleucine in codon 788 of the *RET* gene. No genetic variants of this codon have been known so far. The patient had no other known pathogenic mutations in the examined exons of the *RET* gene. Mutations in exon 13 are thought to lead to a rather mild course of disease. These changes have been assigned to MOD group [9]. According to available databases [12, 13], the synonymous changes have been identified in nine codons of exon 13 (763, 766, 768, 769, 774, 777, 786, 790, and 792). Previously, we have suggested a possible association between synonymous variant p.Leu769Leu polymorphism and a risk of sMTC [15].

Another rare mutation identified in this study refers to exon 14. In this exon, synonymous changes occur in 22 codons [12, 13]. A synonymous genetic variant c.2418 C>T (p.Tyr806Tyr) was found in a 50-year-old woman with a stomach carcinoid tumor. This variant has been identified in populations of South Asia, Africa, and Europe, with the total T allele frequency of 0.0001384. In an analysis of the 1000 Genomes Project database, the same variant has been revealed as a somatic change c.2418C>T (p.Tyr806Tyr), COSM1347814 in large intestine tumor, in a small population in the UK from Indian descent, with the allele frequency of 0.999 for C and 0.001 for T [12, 13]. Gastrointestinal carcinoid tumors develop from neuroendocrine *amine precursor uptake and decarboxylation* (*APUD*) cells. APUD cells constitute a group of apparently unrelated endocrine cells. APUD cells comprise pinealocyte of the pineal gland, C cells of the thyroid, and pheochrome cell of the adrenal medulla. The cells share a common function of secreting a low molecular weight polypeptide hormone [56, 57]. The germinal, c.2418C>T (p.Tyr806Tyr), change should be further examined in patients with gastrointestinal carcinoid tumors and their relatives.

In codon 806 of the *RET* gene, so far, only one germline change has been described, c.2417A>G (p.Tyr806Cys), rs377767419, co-occurring with the p.Val804Met mutation, in a patient with MEN2B [58].

In exon 15, synonymous changes occur only in seven codons. The clinical significance of the synonymous genetic variant c.2673 G>A (p.Ser891Ser), rs201620214 that we discovered in our population is uncertain. This change has been identified in patients with MEN2. The allele frequency for A in the world population is 0.002 [12, 13, 50]. In our study, in one case, the change appeared as a change accompanying the pathogenic mutation in exon 10, but in the other case, except for polymorphic variants, no other pathogenic changes within the examined exons were found. Apart from synonymous changes in this codon, also a pathogenic change, c.2671T>G (p.Ser891Ala), rs75234356, has been described in patients with FMTC, MEN2A, and MTC [14, 59].

Studies on MTC are ongoing to examine carcinogenic mechanisms other than the pathogenic mutations in the *RET* gene. So far, a few mechanisms contributing to MTC development have been described. These are the following:

- mutations in the other genes, e.g., in genes encoding the human RET co-receptors *GFRA1*, *GFRA2*, *GFRA3*, *GFRA4* [60, 61]; RET ligands *ARTN*, *GDNF*, *NRTN PSPN* [62]; or genes encoding the RET downstream effectors, *STAT1*, *AURKA*, *BCL2*, *CDKN2B*, *CDK6*, *COMT*, and *HRAS* [63].
- epigenetic modifications, such as CpG DNA methylation or modifications of histones, which may even be inheritable [64, 65].
- changes in the expression level of the various miRNAs that may be the cause and/or a result of the carcinogenesis [66–70].
- changes in degradative pathway of the RET protein [71–73].

The relationship between synonymous changes in the *RET* gene and the increased risk of MTC is still a subject of controversy. Currently, it is widely accepted that despite the lack of a direct influence of the synonymous variants of the amino acid structure of protein, such changes may influence phenotype and may lead to many diseases. Links between synonymous mutations in different genes and different diseases have recently been proven, and the list is still expanding [25, 74]. Five to 10% of human genes are estimated to contain at least one harmful region because of the synonymous mutations [75]. The current release of the database of deleterious synonymous mutation (dbDSM) collects 1936 synonymous mutation disease (SM disease) association entries, including 1289 SMs and 443 human diseases [76]. By employing cancer-related mutation database, Li et al. indicated that, similarly to nonsense and missense pathogenic mutations, synonymous mutations may also change the dynamical parameters of the corresponding proteins in the TNF-α signaling network and cause a significant increase of the critical dose of TNF-α necessary for cell death [77].

It is impossible to assess the mechanism of action and the potential impact of synonymous variants on the protein function, without the precise testing. Based on in silico studies, it can only be concluded that the four described changes may influence protein synthesis rate, by accelerating it (p.Ser891Ser variant) or slightly slowing it down (the other variants) (Table 3).

**Table 3:** Codon usage comparison

SNP	Codon change	Triplet frequency (H. sapiens)^a^
Cys609Cys	UGC → UGU	12.6 → 10.6
Ile788Ile	AUC → AUU	20.8 → 16.0
Tyr806Tyr	UAC → UAU	15.3 → 12.2
Ser891Ser	UCG → UCA	4.4→ 12.2

^a^Values of codon usage (frequency per thousand) in *Homo sapiens* were taken from the Codon Usage Database [78, 79]

## Conclusions

Rare synonymous changes in the *RET* gene, c.1827C>T (p.Cys609Cys), c.2364C>T (p.Ile788Ile), and c.2673G>A (p.Ser891Ser), were identified in MTC patients and c.2418C>T (p.Tyr806Tyr) in a patient suspected of MEN2. Two of the variants, p.Cys609Cys and p.Ile788Ile, had never been previously described. These findings contribute to a better recognition of the whole range of genetic changes of the *RET* gene and of the involvement of synonymous variants in genetic diversity of this gene.

APUD, amine precursor uptake and decarboxylation; ARTN, artemin; AURKA, aurora kinase A; ATA, American Thyroid Association; ATA-H, RET gene mutations of the high risk; ATA–HST, RET gene mutations of the highest risk; ATA-MOD, RET gene mutations of the moderate risk; BCL2, B cell lymphoma 2; BRCA1/2, breast cancer 1, 2; CAE, carcinoembryonic antigen; CDK6, cyclin-dependent kinase 6; CDKN2B, cyclin-dependent kinase inhibitor 2B; CFTR, cystic fibrosis transmembrane conductance regulator; CHEK2, checkpoint kinase 2; CLA, cutaneous lichen amyloidosis; COMT, catechol-O-methyltransferase; FMTC, familial medullary thyroid carcinoma; GDNF, glial cell line-derived neurotrophic factor; GFRA1–4, GDNF family receptor alpha 1–4; HD, Hirschsprung’s disease; HPTH, hyperparathyroidoism; HRAS, Harvey rat sarcoma viral oncogene homolog; MEN2A, MEN2B, multiple endocrine neoplasia syndromes type 2A, 2B; MTC, medullary thyroid carcinoma; NBS1, Nijmegen breakage syndrome 1; NHLBI, National Heart Lung and Blood Institute; NRTN, neurturin; PHEO, pheochromocytoma; PSPN, persephin; PTC, papillary thyroid carcinoma; RET, rearranged during transfection; SM-disease, synonymous mutation disease; sMTC, sporadic medullary thyroid carcinoma; SNP, single nukleotyde polymorphism; STAT1, signal transducer and activator of transcription 1; TCOF1, treacle ribosome biogenesis factor 1; TNF-α, tumor necrosis factor; TP53, tumor protein P53; UTR, untranslated region

## Electronic supplementary material

ESM 1 (DOCX 15.2 kb)
