# Supplementary material for: Analysis of Newly Identified and Rare Synonymous Genetic Variants in the RET Gene in Patients with Medullary Thyroid Carcinoma in Polish Population
Source: Endocr Pathol. 2017 Jun 24;28(3):198–206. doi: 10.1007/s12022-017-9487-2 (PMC5552825; doi:10.1007/s12022-017-9487-2)
Supplement: Supplementary file 1 — (DOCX 15.2 kb) [file 12022_2017_9487_MOESM1_ESM.docx]

**Supplementary material**

**Analysis of newly identified and rare synonymous genetic variants in the *RET* gene in patients with medullary thyroid carcinoma in Polish population.**

**Maria Sromek, Małgorzata Czetwertyńska, Magdalena Tarasińska, Aneta Janiec-Jankowska, Renata Zub, Maria Ćwikła, Dorota Nowakowska, Magdalena Chechlińska**

PCR primer sequences for exons: 5, 8, 9, 10, 11, 12, 13, 14, 15, 16, 18 and 19 of the *RET* gene*.*

| Exons | Primers | Oligonucleotide sequences | Product size (bp) |
| --- | --- | --- | --- |
| 5 | *RET* 5F | 5' CATCTCGCCTGCACTGACCAAC 3' | 260 |
|  | *RET* 5R | 5' GCCCATGAAGAGCGAGCAC 3' |  |
| 8 | *RET* 8F | 5' GCTGTTCCCTGTCCTTGGGCACTA 3' | 183 |
|  | *RET* 8R | 5' CCTTGGGCGTTTCCAGGGCTTAC 3' |  |
| 9 | *RET* 9F | 5' TGGCGGGGCTCCCACAT 3' | 218 |
|  | *RET* 9R | 5' GAACTGACAGCCCTGGCAACCTCT 3' |  |
| 10 | *RET* 10F | 5' GCGCCC CAGGAGGCTGAGTG 3' | 188 |
|  | *RET* 10R | 5' CGTGGTGGTCCCGGCCGCC 3' |  |
| 11 | *RET* 11F | 5’ ATACGCAGCCTGTACCCAGT 3’ | 457 |
|  | *RET* 11R | 5’ CACAGACTGTCCCCACACAG 3’ |  |
| 12 | *RET* 12F | 5’ GCCTTCTTCCTCCCCTGTCAT 3’ | 216 |
|  | *RET* 12R | 5’ GAGACTCCCCCAGGGGCACTGT 3’ |  |
| 13 | *RET* 13F | 5' CTCTCTGTCTGAACTTGGGC 3' | 238 |
|  | *RET* 13R | 5' TCA CCC TGC AGC AGG CCT TA 3' |  |
| 14 | *RET* 14F | 5' CCCAGG GCCCCTCTCTCCGC 3' | 299 |
|  | *RET* 14R | 5' GTG GGTCAG GGTGTG GCC TG 3' |  |
| 15 | *RET* 15F | 5' TGACCGCTGCTGCCTGGCCAT 3' | 250 |
|  | *RET* 15R | 5' GCTTCCCAAGGACTGCCTGC 3' |  |
| 16 | *RET* 16F | 5' AGG GATAGGGCCTGG GCTTC 3' | 192 |
|  | *RET* 16R | 5' TAACCTCCACCC CAAGAGAG 3' |  |
| 18 | *RET* 18F | 5' AGGGTGCGATGGCTGTGG 3' | 235 |
|  | *RET* 18R | 5' CTGGGAACTCTGAGGGCTAAAACT 3' |  |
| 19 | *RET* 19F | 5' GTGGCACATGGCTTGGAGT 3' | 208 |
|  | *RET* 19R | 5' AGGATAGTGCAGAGGGGACAG 3' |  |
